# Cavitary Lung Lesion as the Initial Presentation of Diffuse Large B-Cell Lymphoma: A Case Report With Review of the Literature

**DOI:** 10.7759/cureus.116499

**Published:** 2026-09-18

**Authors:** Hannah Mckenney, Sidharth Mahajan, Ronald Lands

**Affiliations:** 1 Medicine, University of Tennessee Health Science Center, Memphis, USA; 2 Internal Medicine, University of Tennessee Medical Center, Knoxville, USA; 3 Division of Hematology and Oncology, University of Tennessee Medical Center, Knoxville, USA

**Keywords:** case report, cavitary lung lesion, cavitary pneumonia, diffuse large b-cell lymphoma, extranodal lymphoma, pulmonary lymphoma, secondary pulmonary involvement, sjögren syndrome

## Abstract

Diffuse large B-cell lymphoma (DLBCL) is the most common subtype of non-Hodgkin lymphoma and may rarely present with pulmonary involvement, creating significant diagnostic challenges.

We report the case of a 69-year-old man who presented with a persistent cavitary right lower lobe lung lesion initially treated as recurrent pneumonia. Despite multiple courses of antimicrobial therapy, his symptoms persisted, and extensive infectious etiology evaluation was unrevealing. Bronchoscopic biopsy ultimately demonstrated aggressive DLBCL. Subsequent evaluation revealed small bowel involvement, establishing systemic DLBCL with secondary pulmonary involvement.

This case highlights an uncommon presentation of DLBCL masquerading as refractory pulmonary infection. We also review the previously reported cases of cavitary pulmonary DLBCL. Persistent cavitary lung lesions that fail to respond to appropriate antimicrobial therapy should prompt consideration of malignant etiologies and early tissue diagnosis.

## Introduction

Diagnostic uncertainty is a frequent challenge in clinical practice, particularly when a condition deviates from its expected presentation. Even well-characterized malignancies such as diffuse large B-cell lymphoma (DLBCL) can manifest with unusual features that fall outside of classical teaching. These atypical patterns may delay recognition, especially when initial symptoms overlap with more common conditions. For this reason, clinicians must maintain a broad differential diagnosis and consider serious alternative etiologies even in the absence of typical findings.

Non-Hodgkin lymphoma is the eighth most common cancer in the United States, with DLBCL being the most common subtype [1,2]. DLBCL is an aggressive B-cell non-Hodgkin lymphoma characterized by rapidly growing tumors that commonly involve lymph nodes but may also involve extranodal organs [3]. Pulmonary involvement occurs in approximately 8% of patients with DLBCL according to one study, while cavitary pulmonary lesions are an exceedingly uncommon manifestation that have only been described in case reports [4]. A prolonged interval from symptom onset to diagnostic biopsy in DLBCL is associated with inferior progression-free survival, particularly among patients with a high Ki-67 proliferation index, highlighting the importance of prompt diagnosis and timely initiation of therapy [5].

We report the case of a 69-year-old male who initially presented with a persistent cavitary right lower lobe lung lesion that proved to be an aggressive DLBCL, with subsequent identification of small-bowel involvement establishing systemic disease. This atypical presentation, masquerading as recurrent pneumonia and resistant to multiple courses of antibiotics, illustrates the diagnostic challenges of cavitary pulmonary involvement by DLBCL and underscores the importance of maintaining a broad differential and pursuing timely diagnostic evaluation when presumed infection fails to resolve.

## Case presentation

Patient presentation

A 69-year-old male with a history of hypothyroidism and Sjögren’s syndrome presented to our tertiary care center with approximately four months of progressive dyspnea. He was a hay farmer and reported annual episodes of airway irritation and dry cough during mowing season. Early in the course of his illness, he was evaluated at an urgent care clinic and treated symptomatically with steroids, benzonatate, and guaifenesin without improvement. Approximately two months after symptom onset, he was admitted to an outside hospital for worsening shortness of breath and weight loss. Chest CT at that time demonstrated a large mass-like consolidation within the right lower lobe with central necrosis and adjacent interstitial airspace opacities. He was treated with broad-spectrum antibiotics and discharged. At discharge, he was referred to pulmonology with a repeat CT planned in two months to evaluate for malignancy, although the timing and details of the referral are unclear. Over the following weeks, he completed 10 days of minocycline and levofloxacin followed by 14 days of doxycycline without symptomatic improvement. His respiratory symptoms continued to worsen, with associated nausea, decreased appetite, and continued weight loss. He was subsequently hospitalized for approximately three weeks due to worsening dyspnea and right-sided chest pain.

During this admission, initial CT imaging demonstrated a large mass-like consolidation in the right lower lobe with central necrosis and a 7.8-cm lobulated cavity containing an air-fluid level, associated with mediastinal lymphadenopathy and a large right pleural effusion. Although necrotic malignancy remained in the radiologic differential, infection remained the predominant clinical working diagnosis, and malignancy workup was not pursued. Notably, serum calcium was elevated to 12.9 mg/dL on admission and remained persistently elevated throughout the hospitalization. During this hospitalization, two chest tubes were placed, draining more than two liters of pleural fluid. Pleural fluid analysis revealed a lymphocyte-predominant effusion, with negative cytology for malignancy and negative acid-fast bacilli and fungal cultures. He also received approximately two weeks of inpatient intravenous antibiotic therapy, including vancomycin, complicated by acute kidney injury (AKI). Despite prolonged antimicrobial treatment and pleural drainage, repeat imaging near discharge continued to demonstrate the persistent mass-like right lower lobe consolidation with central cavitation. He was discharged without antibiotics but continued to experience dyspnea, cough, fatigue, and weight loss. One week after discharge, he was evaluated by his primary care provider for persistent symptoms and was found to have hypercalcemia, prompting referral to our institution for further evaluation. Overall, approximately 70 days elapsed from his initial presentation to the outside hospital to his presentation at our institution, during which he underwent multiple hospitalizations and outpatient evaluations without a definitive malignancy workup.

Review of systems was significant for profound fatigue and 35-40 pounds of unintentional weight loss over four months. He denied fever, night sweats, hemoptysis, or new lymphadenopathy.

Clinical findings

On presentation, he was afebrile with a temperature of 96.1°F (35.6°C), normotensive with a blood pressure of 112/75 mmHg, and had a heart rate of 70-75 beats per minute and a respiratory rate of 16 breaths per minute. Oxygen saturation was 95% on room air. His body mass index was 24.4 kg/m² (weight: 88.4 kg, height: 191 cm). He appeared in no acute distress, was conversational, and appeared his stated age. Pulmonary examination revealed unlabored respirations with decreased breath sounds in the right lung, without wheezes or crackles. Cardiovascular examination demonstrated a regular rate and rhythm without jugular venous distension and with adequate perfusion. There was no palpable cervical, supraclavicular, axillary, or inguinal adenopathy. The abdomen was soft, and there was no hepatosplenomegaly. Extremities were atraumatic without edema. Skin examination showed no rashes, bruising, or bleeding.

Evaluation/workup

Chest radiography and CT revealed a large cavitary lesion occupying the majority of the right lower lobe, with associated mediastinal lymphadenopathy and trace pleural air concerning for an early bronchopleural fistula (Figures 1, 2). Enlarged lymph nodes included a 14-mm precarinal node, a 14-mm right paratracheal node, and subcarinal nodes measuring approximately 16 mm. The radiologist emphasized a differential of necrotic malignancy versus cavitary pneumonia and explicitly recommended evaluation for neoplasm.

**Figure 1:**
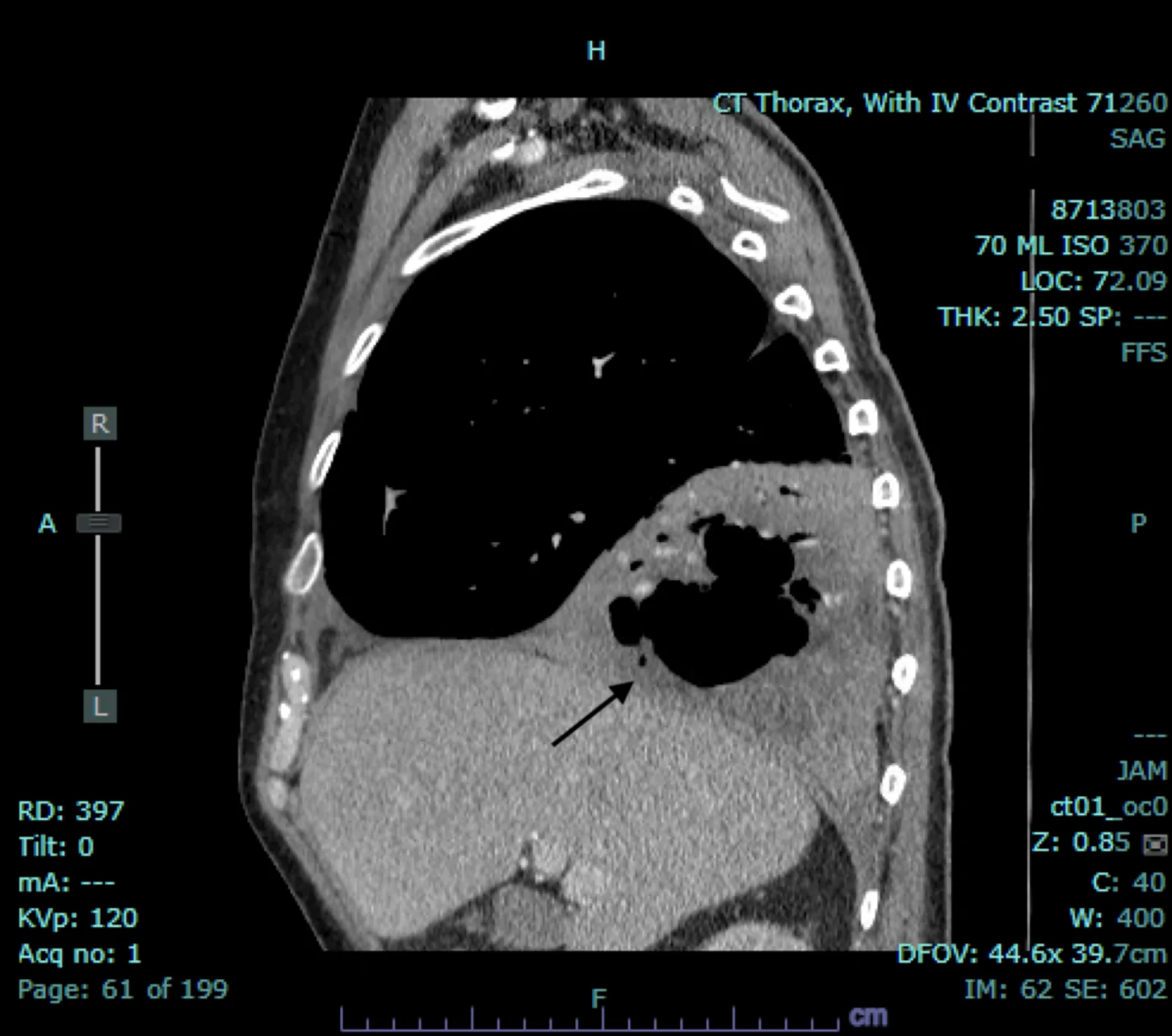
Sagittal CT image demonstrating the right lower lobe cavitary lesion

**Figure 2:**
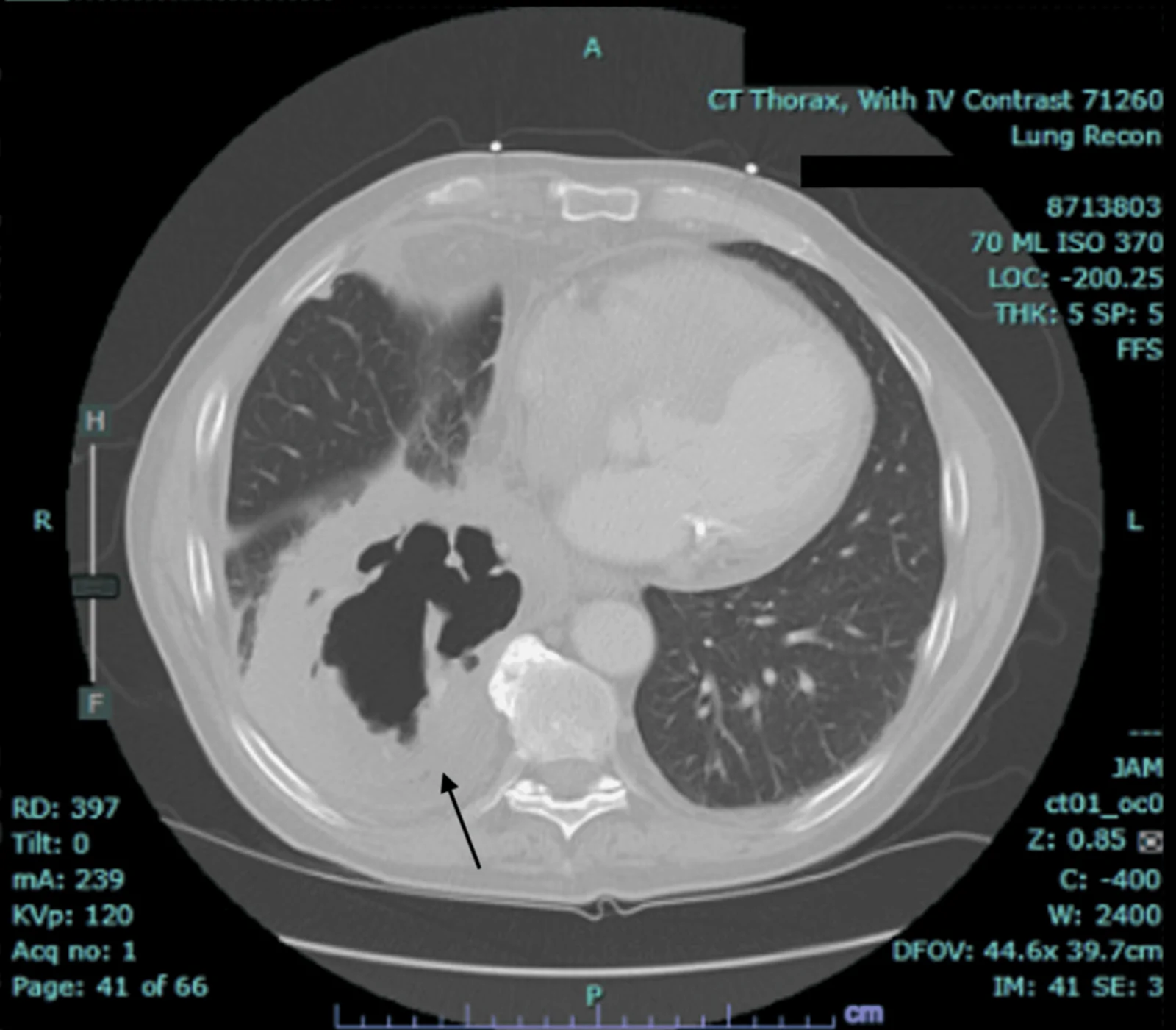
Transverse CT image demonstrating the right lower lobe cavitary lesion

Laboratory evaluation revealed severe hypercalcemia with suppressed parathyroid hormone (PTH) and negative PTH-related peptide, as well as normocytic anemia and AKI attributed to vancomycin, although severe hypercalcemia may have also contributed (Table 1).

**Table 1:** Pertinent laboratory findings WBC: White blood cell; MCV: Mean corpuscular volume; LDH: Lactate dehydrogenase; ESR: Erythrocyte sedimentation rate; CRP: C-reactive protein; PTH: Parathyroid hormone.

Laboratory parameter	Patient value	Reference range
Hemoglobin, initial	12.0 g/dL	12.6–17.7 g/dL
Hemoglobin, nadir	8.7 g/dL	12.6–17.7 g/dL
WBC, initial	10.8 × 10³/µL	3.4–10.8 × 10³/µL
MCV	84 fL	79–97 fL
LDH	180 U/L	100–250 U/L
ESR	46 mm/h	0–30 mm/h
CRP	7.1 mg/dL	0.0–0.5 mg/dL
Albumin, initial	3.0 g/dL	3.4–4.8 g/dL
Creatinine, baseline	~1.1 mg/dL	0.76–1.27 mg/dL
Creatinine, admission	2.79 mg/dL	0.76–1.27 mg/dL
Calcium, peak	13.8 mg/dL	8.8–10.6 mg/dL
PTH	6.66 pg/mL	11.10–79.50 pg/mL
PTH-related peptide	Negative	Negative
1,25-dihydroxyvitamin D	108.0 pg/mL	24.8-81.5 pg/mL

An extensive infectious disease evaluation, including bacterial, mycobacterial, and fungal cultures of pleural fluid, bronchial samples, and blood, was negative. Broad serologic testing for atypical bacterial and fungal pathogens was also unrevealing.

Given the necrotizing appearance of the lesion, antineutrophil cytoplasmic antibody-associated vasculitis was considered. However, serologic testing including perinuclear antineutrophil cytoplasmic antibody, cytoplasmic antineutrophil cytoplasmic antibody, and myeloperoxidase and proteinase 3 antibodies was negative.

Bronchoscopy was performed with lymph node sampling, bronchial washings, and a right lower lobe endobronchial biopsy. The procedure revealed a necrotic-appearing endobronchial lesion in the right lower lobe and enlarged lymph nodes. Rapid on-site evaluation of all sampled lymph nodes was negative. Final cytology from the sampled lymph nodes was negative for malignancy. The endobronchial biopsy of the right lower lobe demonstrated DLBCL, germinal center B-cell (GCB) subtype by Hans criteria. Immunohistochemical staining demonstrated marked B-cell predominance with CD20 and PAX5 expression. The atypical B-cell population was BCL6-positive, while BCL2, CD10, MUM1, CD30, and Cyclin D1 were negative. The Ki-67 proliferation index was approximately 90%, and MYC protein expression was present in approximately 40% of tumor cells. FISH testing for BCL2, BCL6, and MYC rearrangements was within normal limits. Bronchial washings were negative for malignancy and fungal organisms. Staging CT of the abdomen and pelvis demonstrated only indeterminate subcentimeter mesenteric lymph nodes, thought to be reactive, although lymphomatous involvement could not be excluded.

Subsequent clinical course

Three days after discharge, the patient developed new abdominal symptoms prompting further evaluation. A laparotomy with small bowel resection and anastomosis was performed. Pathologic examination of the resected small bowel demonstrated involvement of three separate bowel segments by high-grade B-cell lymphoma consistent with DLBCL​​​​​​. These findings established extranodal lymphoma involving both the lung and small bowel. Hematology/oncology subsequently documented the disease as stage IV DLBCL with pulmonary involvement.

The patient subsequently initiated R-CHOP (rituximab, cyclophosphamide, doxorubicin, vincristine, and prednisone) chemotherapy. Following treatment, he developed severe oral mucositis and dysphagia with poor oral intake, initially tolerating only liquids. His appetite, oral intake, and respiratory symptoms subsequently improved, with associated weight gain. He later developed progressive difficulty with oral intake and ultimately died approximately four months after diagnosis, reportedly following a gastrointestinal bleed.

## Discussion

DLBCL is the most common type of non-Hodgkin lymphoma and accounts for up to 40% of all cases worldwide [2]. It can present as either nodal or extranodal disease. Roughly one-third of cases arise in extranodal sites, involving organs such as the gastrointestinal tract, thyroid, testes, breast, and skin [6]. Pulmonary involvement is less common. 

Primary pulmonary lymphoma is typically defined as lymphoma limited to the lungs at the time of diagnosis, with no evidence of disease outside the thorax at diagnosis or during the following three months. Ipsilateral hilar lymph node involvement is still considered consistent with a primary pulmonary origin [7]. In this case, although the disease initially appeared to be confined to the right lung, further evaluation within two weeks of presentation revealed small bowel involvement confirmed by biopsy. This finding supports a diagnosis of systemic DLBCL with secondary pulmonary involvement rather than primary pulmonary lymphoma.

Pulmonary manifestations of non-Hodgkin lymphoma are most commonly reported as nodules, masses, consolidations, ground-glass opacities, and lymphangitic spread. Cavitary pulmonary involvement is a rare manifestation of lymphoma but has been reported in association with DLBCL [8,9]. Table 2 summarizes previously reported cases of DLBCL presenting with cavitary lung lesions and was compiled by updating the review published by Uppal et al. [9] with additional published cases identified through a targeted PubMed search through July 2026 using combinations of the terms including "diffuse large B-cell lymphoma," "DLBCL," "pulmonary," "lung," and "cavitary."

**Table 2:** Published cases of pulmonary diffuse large B-cell lymphoma presenting as cavitary lung lesions

Case	Age	Gender	Chest CT
Zuo et al., 2026 [10]	60	Male	Multiple cavitary masses
Jain et al., 2025 [11]	65	Male	Single cavitary mass
Uppal et al., 2023 [9]	50	Female	Multiple cavitary masses
Lin et al., 2022 [12]	80	Male	Single cavitary mass with central cavitation
Pathak et al., 2022 [13]	61	Male	Multiple cavitary masses
Hibino et al., 2020 [14]	71	Male	Single cavitary mass
Insiripong et al., 2018 [15]	58	Male	Single cavitary mass with air-fluid level, pleural effusion
Matsumoto et al., 2015 [16]	80	Male	Single cavitary mass with fluid, pleural effusion
Yamane et al., 2011 [17]	68	Female	Single cavitary mass
Miyahara et al., 2001 [18]	27	Male	Single cavitary mass
Mondschein *et al., *1993 [19]	32	Male	Multiple cavitary masses

The management of B-cell lymphomas is determined by disease stage, biologic behavior (indolent versus aggressive), and molecular subtype. DLBCL is an aggressive lymphoma; however, outcomes can be favorable with combination chemotherapy, although cure rates remain variable. Relevant to this case, the established frontline treatment regimen for germinal center subtype DLBCL is R-CHOP every 21 days; however, treatment intensity and duration should be individualized according to disease stage, prognostic risk, patient fitness, and relevant molecular and pathological features [20].

The patient’s history of Sjögren’s syndrome represents a recognized risk factor for lymphoproliferative disease and provides relevant clinical context for the subsequent diagnosis of B-cell lymphoma. The risk of developing non-Hodgkin lymphoma is approximately 7- to 19-fold higher in patients with Sjögren's syndrome compared with the general population. In one cohort study of patients with Sjögren's syndrome-associated non-Hodgkin lymphoma, mucosa-associated lymphoid tissue (MALT) lymphoma was the predominant histologic subtype, accounting for approximately 80% of cases (73/92), whereas DLBCL accounted for approximately 13% (12/92) [21]. Additionally, MALT lymphoma can undergo histologic transformation to DLBCL, which represents another possible mechanism of disease progression in this case [22].

From a diagnostic standpoint, cavitary pulmonary lesions have a broad differential diagnosis that includes both infectious and malignant etiologies. In a retrospective CT-based cohort of patients with multiple cavitary lung lesions, benign processes accounted for 72.6% of cases, while malignant etiologies comprised 27.4%. Within the benign group, tuberculosis was the predominant cause, representing 49.0% of all lesions, followed by lung abscess (8.8%), mycetoma (5.9%), septic emboli (2.9%), atypical mycobacteriosis (2.0%), and atypical aspergillosis (2.0%), with less common etiologies including fusariosis and granulomatosis with polyangiitis (each 1.0%). Among malignant etiologies, metastatic extrapulmonary malignancies accounted for 19.6% of all lesions, whereas primary pulmonary neoplasms accounted for 7.8% [23].

Overall, these data highlight that infectious etiologies, particularly tuberculosis, predominate in the differential diagnosis of cavitary lung lesions, while malignancy remains a substantial but less frequent cause. This patient underwent multiple hospitalizations and outpatient evaluations over 70 days before presenting to our institution, during which infection remained the predominant clinical working diagnosis, and a definitive malignancy workup was not pursued. In retrospect, failure to improve despite prolonged antimicrobial therapy, repeatedly negative infectious studies, constitutional symptoms, and unexplained hypercalcemia should have prompted workup of a noninfectious etiology, including malignancy.

This case demonstrates the challenges of diagnosing DLBCL when it presents outside of its typical pattern. Systemic DLBCL with secondary pulmonary involvement is an uncommon manifestation and may not be immediately considered in the initial differential diagnosis when pulmonary findings predominate. However, a history of autoimmune disease, particularly Sjögren’s syndrome, should increase clinical awareness of the risk of lymphoproliferative disease. In patients receiving immunosuppressive therapy, the broader differential should also include therapy-associated lymphoproliferative disorders. By presenting this case, we aim to highlight the need for heightened awareness of atypical extranodal presentations of DLBCL, particularly in patients with predisposing conditions, and to reinforce the importance of obtaining tissue diagnosis when a cavitary pulmonary lesion persists despite appropriate antimicrobial therapy and persistently unrevealing infectious evaluations.

## Conclusions

DLBCL may rarely present as a cavitary pulmonary lesion and closely mimic persistent or recurrent infection, creating significant diagnostic challenges. This case of systemic DLBCL with secondary pulmonary involvement highlights the importance of maintaining a broad differential diagnosis when cavitary lung lesions fail to respond to appropriate antimicrobial therapy. In patients with persistent cavitary pulmonary lesions, constitutional symptoms, unexplained laboratory abnormalities such as hypercalcemia or anemia, and risk factors for lymphoproliferative disease, including Sjögren’s syndrome, malignancy should be considered, and tissue diagnosis should be pursued when infectious evaluation fails to establish an alternative diagnosis.
